# Emergence of resistance against direct acting antivirals in chronic HCV patients: A real-world study

**DOI:** 10.1016/j.sjbs.2021.12.044

**Published:** 2021-12-24

**Authors:** Abdul Majid, Sanaullah Khan, Sami Siraj, Sumbal Haleem, Najib ul Haq, Riaz Ullah, Essam A. Ali, Adeela Mustafa, Hidayat Hussain, Muhammad Sohaib

**Affiliations:** a Department of Zoology, Kohat University of Science and Technology, Kohat, Pakistan; b Department of Zoology, University of Peshawar, Pakistan; c Institute of Basic Medical Sciences, Khyber Medical University, Pakistan; d Peshawar Medical College, Riphah International University, Pakistan; e Department of Pharmacognosy, College of Pharmacy, King Saud University, Riyadh 11451, Saudi Arabia; f Department of Pharmaceutical Chemistry, College of Pharmacy, King Saud University, Riyadh 11451, Saudi Arabia; g Community Medicine Department, Khyber Medical College Peshawar, Pakistan; h Department of Bioorganic Chemistry, Leibniz Institute of Plant Biochemistry, Weinberg 3, D-06120 Halle (Salle), Germany; i Department of Soil Science, College of Food and Agricultural Sciences, King Saud University, 12 Riyadh 11451, Saudi Arabia

**Keywords:** HCV, **Hepatitis C**, DAA, Direct Acting Antiviral, HCV, Sofosbuvir, Ribavirin, DAA, Interferon

## Abstract

Interferon/Ribavirin therapy has been replaced by Direct Acting Antivirals (DAAs) due to emergence of Resistance Associated Variants (RAVs) and decrease Sustain Virologic Response (SVR). Current study investigated treatment response of Sofosbuvir and Ribavirin in chronic HCV patients. Total 256 HCV patients with genotype 1a, 2 and 3a received sofosbuvir/ribavirin according to international standards. HCV RNA presence in serum was used as marker for end treatment response (ETR) and sustain virologic response after 24 weeks of treatment (SVR24) in each case. Response to treatment with SOF + RBV was found statistically significant among different HCV genotypes (GT) as out of 47 HCV GT1 patients 42(89.36%) resulted into good ETR but 4(9.52%) of these relapsed and 5(10.63%) led into virologic failure. 5(100%) HCV GT2 patients resulted into SVR24 whereas, out of 204 HCV GT3 patients 194(95.69%) achieved good ETR however, 8(4.12%) of these relapsed and 10(4.90%) resulted in to virologic failure. Efficacy of therapy was found non-significant in treatment naïve and treatment experienced patients as in this study out of 145 treatment naïve patients 139(95.86%) achieved good ETR where 4(2.87%) relapsed while 6(4.13%) led into virologic break through on the other hand among 111 treatment experienced patients 102(91.89%) resulted into good ETR but 8(7.84%) relapsed whereas 9(8.10%) lead into virologic failure. Current study also propose that various liver and spleen complications/liver cirrhosis are related to response of HCV patients to SOF + RBV therapy whereas, variables like old age, gender is not compromising treatment response to DAAs therapy. Various mild side effects encountered by patients during treatment were fatigue, insomnia, headache, nausea, burning body, diarrhea, cough. Overall, this study reported 89.45% efficacy of SOF + RBV regime in chronic HCV Pakistani patients. Current study suggests hunting for possible reasons of resistance so that SOF + RBV therapy may not share the same fortune as previous therapies in near future.

## 1 Introduction

Infection with hepatitis C virus (HCV) is a worldwide burning health concern of the day and about 70 million people are harboring the infection. Approximately one-third of the chronically infected population eventually lead into hepatocellular carcinoma (HCC), liver cirrhosis and is a cause of deaths for more than 350,000 people every year (Li et al., 2017). HCV genome variate due to lack in proof reading of polymerase and classified into six major genotypes, each respond differently to antiviral therapy (Tapper and Afdhal, 2013, Zeuzem et al., 2014).

Until recent, till the advent of direct acting antivirals (DAAs) introduced in 2011, the standard of treatment for all HCV patients was 24–48 weeks treatment with interferon/PEGylated interferon (PEG-IFN) with or without ribavirin (RBV) (Ghany et al., 2009). This treatment had revealed very adverse side effects i.e., depression, cytopenia, flue-like conditions, lethargy, and painful subcutaneous weekly injections (Chung, 2012). Though IFN regimes resulted in satisfactory SVR in the start but later on many resistant associated variants were reported (Feld et al., 2015, Patiño-Galindo et al., 2016). Various DAAs are available for the treatment of HCV but nucleotide inhibitor of HCV NS5B polymerase, an oral drug sofosbuvir (SOF) and RBV has pan-genotypic effect (Jacobson et al., 2013, Lawitz et al., 2013). DAAs resulted in improved SVR nearly 40% in patients infected with HCV but due to the advent of swift resistance to this monotherapy its applications were hampered. Although with combination therapy (Protease inhibitors, PEGylated Interferon and RBV) SVR rates heightened but it revealed toxic consequences especially in patients with liver cirrhosis (Jacobson et al., 2013, Poordad et al., 2013, Stedman, 2014). Viral persistence in infected individuals and reduced vulnerability to therapy might lead to mutation in HCV genome.

Rates of SVR among patients treated with SOF and RBV ranges from 92% to 99% but treatment history may affect the outcomes of treatment (Jacobson et al., 2013, Zeuzem et al., 2014). The only mutation which has been detected during trials studies is S282T and other mutations which have been associated with SOF and RBV treatment are V321A and L159F. However, no clear linkage has been established among these mutations and resistivity to SOF. During in vitro studies neither the V321A mutation and nor the L159F mutation conferred resistance to SOF (Zeuzem et al., 2014).

This study was conducted in chronic HCV Pakistani patients infected with genotypes 1, 2 and 3 to find out the response of treatment naïve and treatment experienced patients i.e., those who had already received interferon-based regime but were either not-responding or relapsed to interferon-based therapies.

## 2 Materials and methods

### 2.1 Study design

**Fig. 1:**
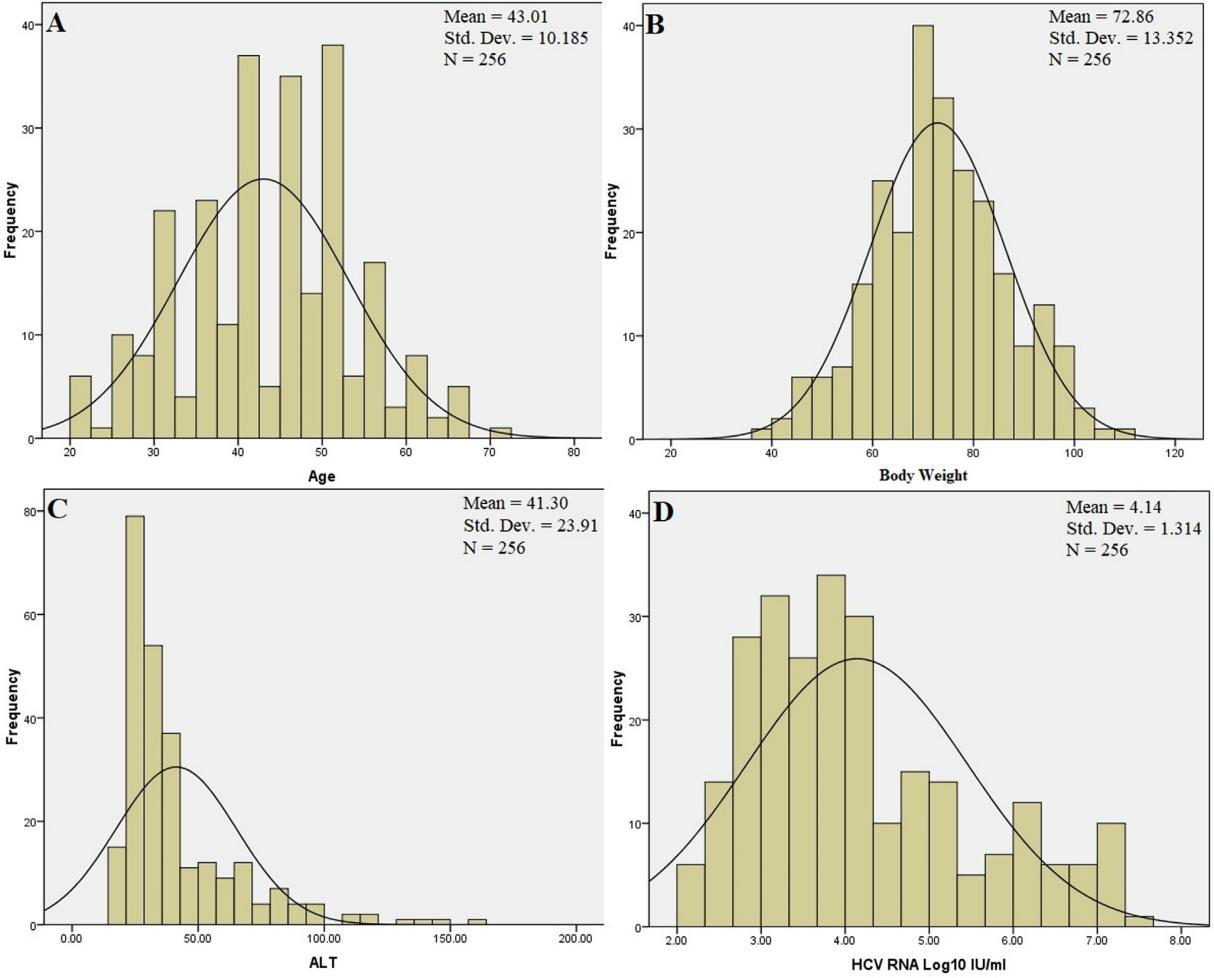
Histogram showing (A) Age of the study population. (B) Body weight was measured for the study participants. (C) ALT values of the patients. (D) Levels of HCV RNA Log10 IU/ml circulating in blood.

This was a random study, patients of either gender with age range from (20–70) years having chronic HCV infection with genotype, 1, 2, 3 and HCV RNA circulating in their serum were included in this study “Fig. 1”. Clinical characteristics i.e., body weight, ALT values, HCV RNA Log10 IU/ml are shown in “Fig. 1” through histogram. Patients were enrolled in this study regardless of their previous treatment history. Both treatment naïve patients who had not received any previous treatment and patients already treated with interferon-based therapies but were unable to attain SVR or were not responding were included in this study. All those candidates were also included in this research project who started interferon treatment but discontinued because of intolerability viz severe adverse side effects. Informed oral consent of each participant of the study was taken “informed, oral consent” was sufficient to obtain institutional approval as (majority of the studied population was illiterate where written consent was not possible that’s why only oral consent was obtained).

Participants were asked for previous interferon therapy and were screened for HCV RNA circulating in their serum at the time of enrollment. Enrolled participants were recommended sofosbuvir oral dose of 400 mg/day in combination with ribavirin oral dose twice daily. Ribavirin dose was regulated according to body weight, patients having mass <75 kg were given 1000 mg/day and 1200 mg/day for those having mass ≥75 kg. Patients infected with HCV genotype 1 and 2 received 12 weeks’ administration of sofosbuvir and ribavirin. According to the results of FUSION phase 3 study which strongly recommended that by increasing the time period of treatment from 12 weeks to 24 weeks in case of HCV genotype 3 could be beneficial (Jacobson et al., 2013). Patients infected with HCV genotype 3 were treated with sofosbuvir and ribavirin for 24 weeks.

A total of 287 patients were enrolled in the present study who were positive for HCV core region antigen through ELISA. ELISA was performed as per manufacturer protocol (QuickTiter™ HCV Core Antigen ELISA Kit, Germany). HCV RNA from the serum samples of ELISA positive HCV patients was isolated according to instructions of the manufacturer manual (FavorPrep Viral Nucleic Acid Extraction Kit I, Taiwan). The concentration and purity of the DNA obtained were determined using the NanoDrop spectrophotometer 2000 (Thermo Scientific, USA) (Christodoulakis et al., 2008). HCV RNA was quantified by real-time PCR using a BioRad MyiQ Single-Color real-time PCR detection system (Bio-Rad, CA) as described by (Bull et al., 2016, Tu et al., 2008).

The prime effective end point was (SVR24) sustained virologic response at 24 weeks after the end of treatment. The prime effective end point was defined as clearance of circulating HCV RNA in serum of the patient through PCR.

### 2.2 Study approval

Present study was approved by Institutional Review Board, Prime Foundation Pakistan. The investigators, participating institutions and sponsor agreed to maintain the confidentiality of the data. All authors vouch for the completeness and accuracy of the data and data analysis and for the fidelity of this report. The manuscript was prepared by the first author and with the input from all the authors.

### 2.3 Statistical analysis

The data was statistically analyzed using SPSS version 23. The difference between proportions were compared through 2-proportion Z-test, Column statistic and Chi-square test. The difference between proportions were considered to be statistically significant if the P-value was less than or equal to 0.05.

## 3 Results

### 3.1 Patients

**Fig. 2:**
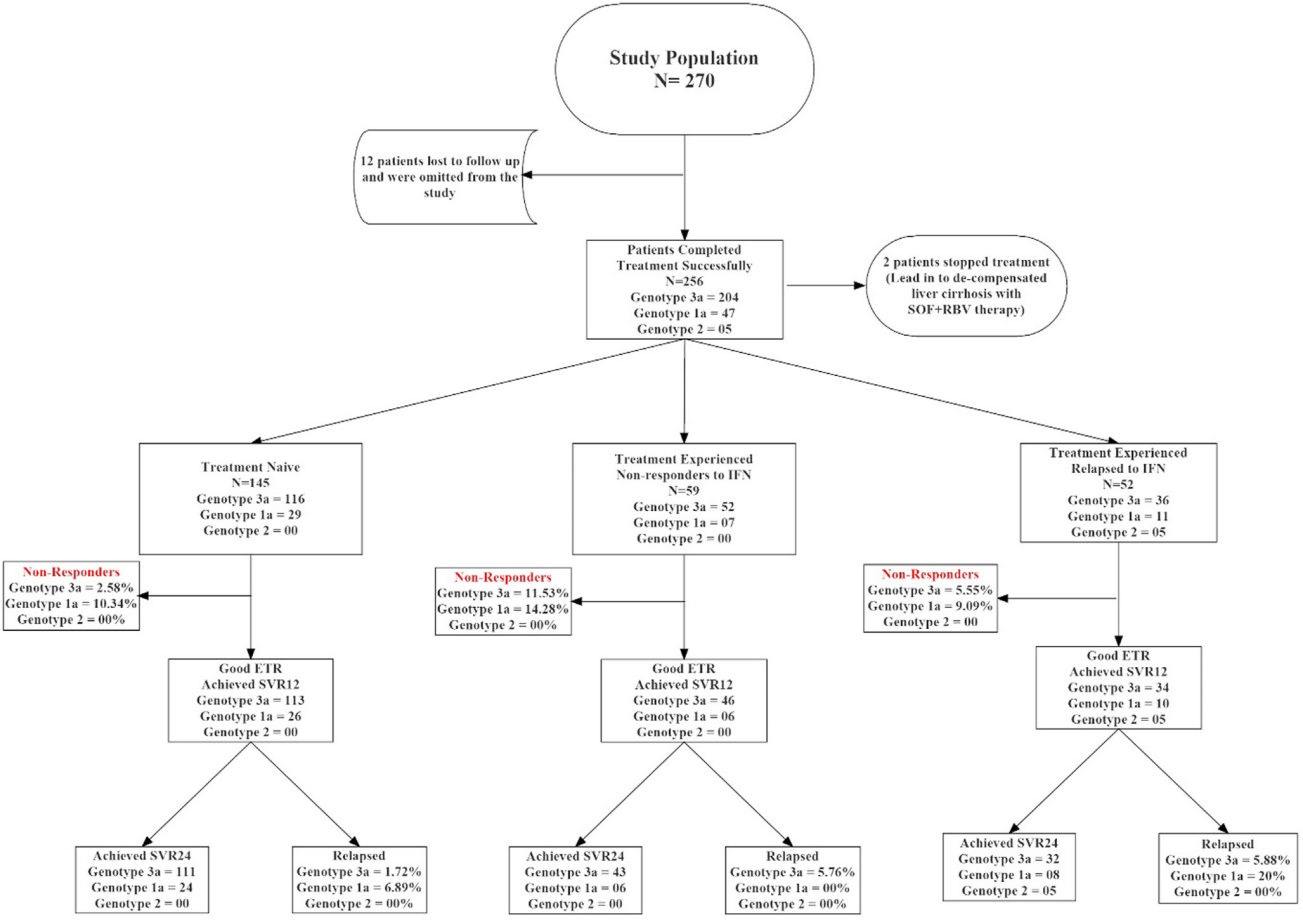
Flowsheet diagram showing overall picture of the study population.

**Fig. 3:**
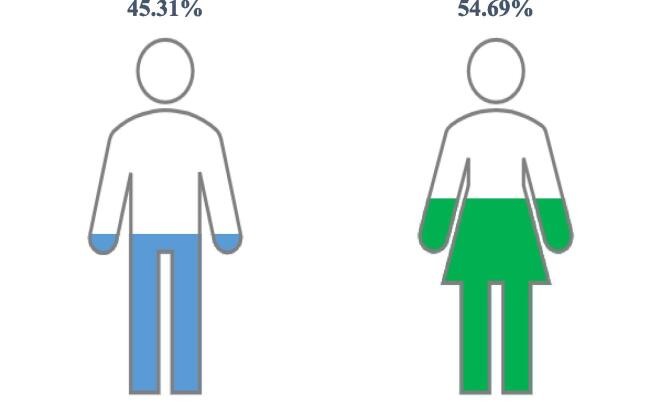
Showing gender wise distribution of population.

Among 17 patients HCV RNA was not confirmed through PCR, they were excluded from the study. Afterwards HCV genotyping was performed for each patient. Later on, 14 patients lost to follow up and were omitted from the study “Fig. 2”. Out of 256 patients, most common genotype reported was genotype 3 (GT3) 204(79.68%) followed by GT1 47(18.35%), and 5(1.95%) patients were infected with GT2. The demographic and baseline clinical characteristics of the studied population are given in “Table 1”. Overall, 90(44.11%) of the studied population were male and 114(55.88%) were female “Fig. 3”. Mean age of the studied population was recorded 43.01 years ranging from 20 to 70 years. Mean weight of the participants of the study was 72.88 kg ranging from 36 to 111 kg “Fig. 1”.

**Table 1:** Baseline characteristics of the study population.

Characteristics	HCV genotypes			Overall
	GT3a	GT1a	GT2	N
**Patients Included (%)**	204(79.68)	47(18.35)	5(1.95)	**256**
**Male n (%)**	90(44.11)	21(44.68)	5(100)	**116(45.31)**
**Female n (%)**	114(55.88)	26(55.31)	00(00.00)	**140(54.68)**

*Age*				
Mean	42.55	44.47	48.2	**43.01**
Range	20–66	22–70	43–55	**20**–**70**

*Body Weight*				
Mean	73.04	70.98	84.4	**72.88**
Range	36–105	42–111	68–100	**36**–**111**

*HCV RNA Log_10_ IU/ml*				
Median	3.9	3.3	3.3	**3.8**
Range	2.10–7.10	2.10–7.10	2.70–4.10	**2.10**–**7.60**

*ALT*				
Mean	40.78	43.32	43.8	**41.3**
Range	18–161	20–147	27–68	**18**–**161**
**Cirrhosis n (%)**	23(11.27)	1(2.12)	0(00)	**24(9.38)**
**HCC n (%)**	2(0.98)	0(00)	0(00)	**2(0.78)**
**Treatment Naïve n (%)**	116(56.86)	29(61.70)	0(00)	**145(56.64)**
**Treatment Experienced n (%)**	88(43.14)	18(38.30)	5(100)	**111(43.36)**
**Non-Responder to IFN n (%)**	52(59.09)	7(38.89)	0(00)	**59(53.15)**
**Relapsed to IFN n (%)**	36(40.91)	11(61.11)	5(100)	**52(46.85)**

*Liver*				
Fatty Liver	17(8.33)	4(8.51)	0(00)	**21(8.20)**
Hepatomegaly	16(7.84)	2(4.26)	0(00)	**18(7.03)**

*Spleen*				
Splenomegaly	22(10.78)	6(12.77)	1(20)	**29(11.33)**
**HBV Carrier**	1(0.49)	0(00)	0(00)	**1(0.39)**

GT (Genotype), HCC (Hepatocellular Carcinoma).

**Fig. 4:**
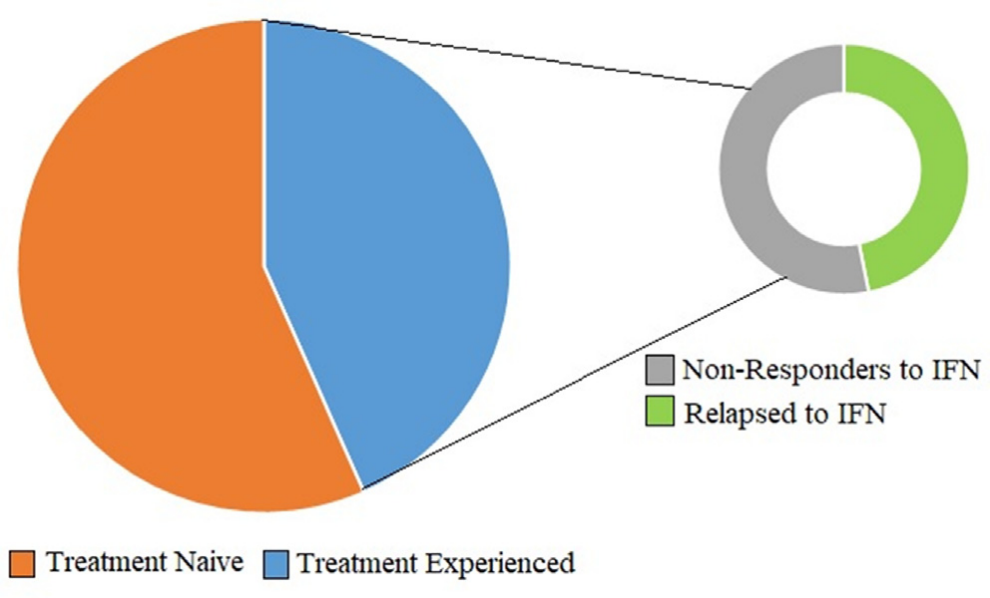
Treatment history of patient included in the study.

Overall population was divided into two major groups those who had previous HCV treatment history 111(43.36%) and treatment naïve patients 145(56.64%) who have not been through any HCV treatment before. Among treatment experienced patients 52(46.84%) had relapsed to interferon/PEGylated interferon therapy having a virologic break through while the remaining 59(53.15%) were not responders to interferon/PEGylated interferon therapy “Fig. 4”.

### 3.2 Effectivity

This study reported 89.45% efficacy of SOF + RBV regime in chronic HCV patients. The effectivity of SOF + RBV was measured by determining circulating HCV RNA levels. In “Table 2” are shown results of HCV RNA levels after completion of treatment with Sofosbuvir. Out of total 256 patients 229 had undetectable HCV RNA levels while 27 patients who failed to achieve sustain virologic response (SVR24) had mean of 4.47 HCV RNA Log10 IU/ml circulating in blood. Among 47 patients infected with HCV GT1 42(89.36%) achieved good end treatment response (ETR) but 4(9.52%) of these patients relapsed and only 38(90.47%) achieved sustain virologic response (SVR24) after 24 weeks of treatment while 5(10.63%) led to virologic failure. HCV patients infected with GT2 5(100%) resulted into SVR24. Patients infected with HCV GT3 received SOF + RBV for 24 weeks, among 204 patients 194(95.09%) resulted into good ETR but 8(4.12%) of these participants failed to attain SVR24 and relapsed while 10(4.90%) were unable to clear viral load and resulted into virologic break through. The results were compared statistically, and the data was found significant “Table 2”.

**Table 2:** Showing HCV RNA levels after sofosbuvir therapy.

Patients Not-responding to Sofosbuvir Therapy	HCV RNA Log10 IU/ml
N	27
Mean	4.47
Std. Deviation	1.001
Minimum	2.70
Maximum	6.20
Patients with Good End Treatment Response	Undetectable HCV RNA Levels
N	229

Among 145 treatment naïve patients 139(95.86%) had good ETR but among these patients 4(2.87%) failed to achieve SVR24 and relapsed while 6(4.13%) of the patients were not responding to treatment. Out of 111 patients with previous treatment history 102(91.89%) had good ETR nonetheless 8(7.84%) relapsed whereas 9(8.10%) resulted into virologic failure the data was statistically non-significant “Table 3”.

**Table 3:** Association of different variables with treatment response of patients to SOF + RBV therapy.

Variables	N256	Good ETR	CI 95%	P Value	Relapse	CI 95%	P Value	NR	CI 95%	P Value
*Treatment History*										
Treatment Naïve	**145**	139	−0.0073–0.1473	0.0759	04	−0.0132–0.1132	0.1209	06	−0.0258–0.1058	0.2337
Treatment Experienced	**111**	102			08			09		

*Gender*										
Male	**116**	109	−0.0610–0.0545	0.9135	07	−04041–0.0804	0.5164	07	−0.0545–0.0610	0.9135
Female	**140**	132			05			08		

*Age*										
**≤**40	**108**	103	−0.0369–0.0795	0.4742	01	−0.11900–0.02101	0.0135	05	−0.0795–0.0369	0.4742
˃40	**148**	138			11			10		

*HCV Genotypes*										
GT 1a	**47**	42		0.002	04		0.003	05		0.002
GT 2	**05**	05			00			00		
GT 3a	**204**	194			08			10		

*ALT U/L*										
Less than or equal to 50	**199**	190	−0.0152–0.1352	0.1179	8	−0.0258–0.1058	0.2337	9	−0.023–0.123	0.1795
Above 50	**57**	51			4			6		

*HCV RNA log10 IU/ml*										
Less than or equal to 4	**142**	136	−0.0258–0.1058	0.2337	05	−0.0332–0.0932	0.3521	06	−0.0258–0.1058	0.2337
Above 4	**114**	105			07			09		
*Cirrhosis*										
Cirrhotic	**22**	15	0.1513–0.3687	0.0001	7	0.3308–0.5692	0.0001	7	0.1847–0.3953	0.0001
Non-Cirrhotic	**234**	226			5			8		

*Liver & Spleen Complications*										
Normal	**204**	188		0.0001	07		0.0001	09		0.0001
Splenomegaly	**24**	18			03			03		
Hepatomegaly	**03**	03			00			00		
Hepatosplenomegaly	**06**	04			01			01		
Enlarged Fatty Liver	**19**	16			01			02		

NR (Not Responding), ETR (End Treatment Response), GT (Genotype).

In the study population 116(45.35%) participants were male while 140(54.68%) were female “Fig. 3”. The response of male and female population to treatment was found statistically non-significant. As among male participants 109(93.96%) resulted into good ETR where 7(6.42%) relapsed and 7(6.03%) were not responding to therapy. In female population 132(94.28%) achieved good ETR while 5(3.78%) relapsed and 8(5.71%) were not responding to DAAs therapy “Table 2”.

### 3.3 Drug side effects

**Fig. 5:**
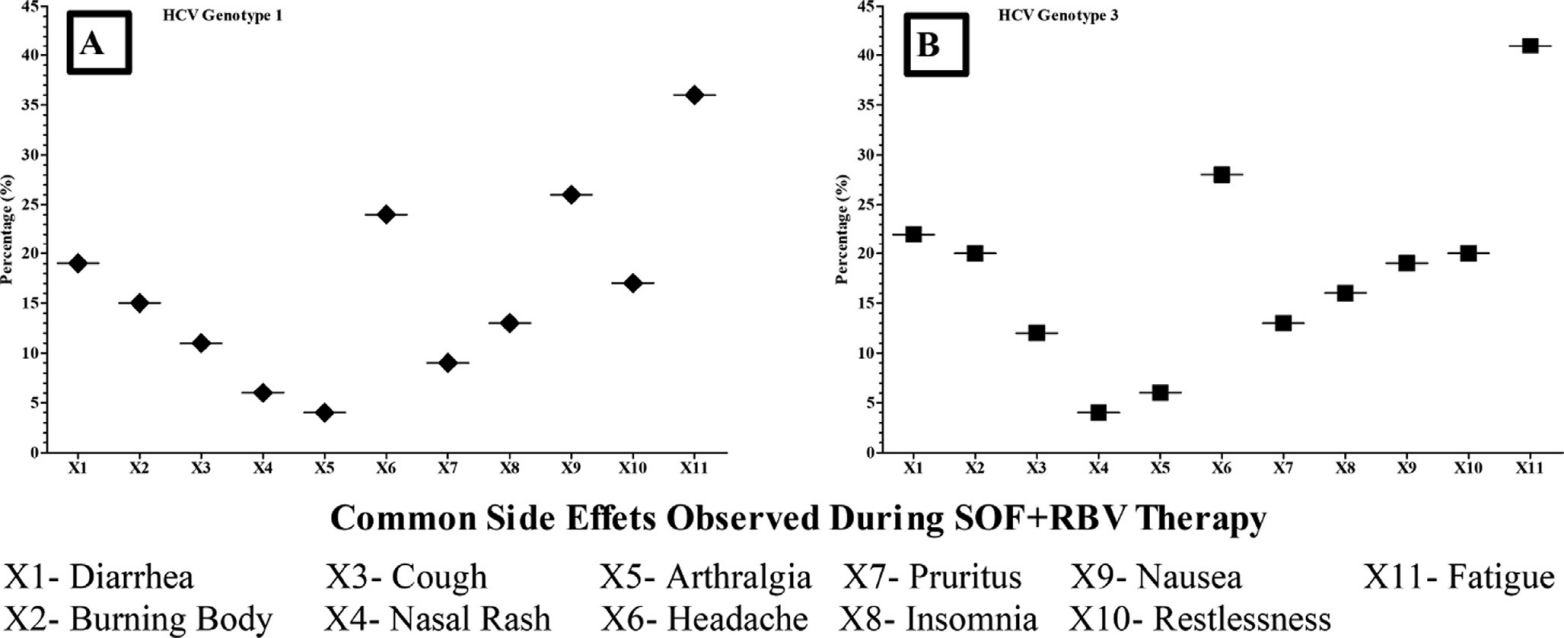
Showing various common side effects experienced during therapy.

Mild side effects in the study participants observed during SOF + RBV therapy were fatigue, nausea, insomnia, headache, diarrhea, burning body. Detail of side effects is shown in “Fig. 5” as were also reported by other studies (Bull et al., 2016, Zeuzem et al., 2014). Two patients having compensated liver cirrhosis stopped treatment as they developed decompensated liver cirrhosis with the use of SOF + RBV.

## 4 Discussion

Direct acting antivirals come into market for HCV treatment in 2011, before DAAs only treatment strategy for HCV treatment was Interferon therapy. IFN therapy was compromised because it faced two challenges. First the treatment was painful, very lengthy and had many side effects. Second very important challenge was appearance of huge number of resistant associated variants. Keeping in view the fate of IFN therapy this study focused on response of chronic HCV Pakistani patients to Sofosbuvir (a nucleotide inhibitor) and Ribavirin combination therapy. Keeping in view the availability of direct acting antivirals in Pakistan Sofosbuvir was selected for chronic HCV patients’ treatment as it was the only available DAA at the time of execution of this study. Genotyping results show that highest number 204(79.68%) of patients in Pakistan are infected with HCV genotype 3a, while a small portion of the population 5(1.95%) was infected with HCV genotype 2, which is similar to studies published somewhere else (Jamil et al., 2018, Khan et al., 2019). In this study the overall response of chronic HCV patients to SOF + RBV reported was 229(89.45%) parallel reports have been seen in many studies (Dalgard et al., 2017, Mushtaq et al., 2020, Zeuzem et al., 2014).

Most noticeable HCV genotypes studied in this research work were HCV GT1a and HCV GT3a. Results of this project showed that the response of HCV GT1a was lower 42(89.36%) as compared to HCV GT3a which was 194(95.09%). The data is statistically significant as P-value calculated was less than 0.05 (Ghany et al., 2011, Noell et al., 2015). This study was consisted of two major groups i.e., in treatment naïve patients the efficacy of DAAs therapy was reported 139(95.86%) which is numerically a little higher than treatment experienced patients 102(91.89%) (Dalgard et al., 2017, Mushtaq et al., 2020, Zeuzem et al., 2014). Study population was comprised of both genders. However, there was no significant difference reported in this study regarding the response of both genders i.e. (male 109(93.96%) and female 132(94.28%)) to SOF + RBV treatment.

The results of this study also suggest that age does not affect the response of patients to SOF + RBV treatment. In both age groups i.e., patients with age 40 years or less than 40 years the response to treatment was recorded 103(95.37%) and patients with age above 40 years the response was 138(93.24%) which as statistically non-significant as P-value less than 0.05. Patients having ALT values equal to or less than 50 U/L and patients having ALT values above 50 U/L responded in similar way to SOF + RBV therapy. Viral load circulating in the patients blood has no effect on the response of patients to DAAs therapy as the data was found statistically non-significant according to the findings of this study.

Total numbers of patients with liver cirrhosis enrolled in this project were 24(9.83%). After 6 weeks of treatment two patients having liver cirrhosis lead into decompensated liver cirrhosis and we have to stop their treatment and were excluded from the study. The rest of patients with liver cirrhosis completed their treatment successfully and their response to SOF + RBV therapy was compared with the response of patients with normal liver. The results showed that there was a significant difference P > 0.05 among the response cirrhotic and non-cirrhotic patients to therapy (Forde and Bhattacharya, 2017, Mushtaq et al., 2020, Noell et al., 2015).

Two chronic HCV patients having hepatocellular carcinoma completed their treatment but could not achieved end treatment response. Our findings also suggest that certain liver abnormal conditions such as hepatomegaly, hepatosplenomegaly may affect the response of HCV patients to DAAs therapy. The most common complications or side effects encountered the patients of this study were fatigue, headache, diarrhea, burning body, nausea, restlessness, insomnia (Zeuzem et al., 2014).

In conclusion, oral regimen of SOF + RBV offer interferon free therapy, which is effective against HCV genotype 1, 2 and 3. Current study reported first time resistance to SOF + RBV treatment in Pakistan, which warrant further detail research to search for possible reasons of resistance otherwise SOF + RBV therapy may also encounter the same fortune as previous therapies in near future.

## Ethical Statement

Present study was approved by Institutional Review Board, Prime Foundation Pakistan.

## Declaration of Competing Interest

The authors declare that they have no known competing financial interests or personal relationships that could have appeared to influence the work reported in this paper.
